# Electroporation Knows No Boundaries: The Use of Electrostimulation for siRNA Delivery in Cells and Tissues

**DOI:** 10.1177/1087057115579638

**Published:** 2015-09

**Authors:** Christin Luft, Robin Ketteler

**Affiliations:** 1 MRC Laboratory for Molecular Cell Biology, University College London, London, UK

**Keywords:** electroporation, RNA interference, high-throughput screening, cell transfection

## Abstract

The discovery of RNA interference (RNAi) has enabled several breakthrough discoveries in the area of functional genomics. The RNAi technology has emerged as one of the major tools for drug target identification and has been steadily improved to allow gene manipulation in cell lines, tissues, and whole organisms. One of the major hurdles for the use of RNAi in high-throughput screening has been delivery to cells and tissues. Some cell types are refractory to high-efficiency transfection with standard methods such as lipofection or calcium phosphate precipitation and require different means. Electroporation is a powerful and versatile method for delivery of RNA, DNA, peptides, and small molecules into cell lines and primary cells, as well as whole tissues and organisms. Of particular interest is the use of electroporation for delivery of small interfering RNA oligonucleotides and clustered regularly interspaced short palindromic repeats/Cas9 plasmid vectors in high-throughput screening and for therapeutic applications. Here, we will review the use of electroporation in high-throughput screening in cell lines and tissues.

## Introduction

**Figure 1.**
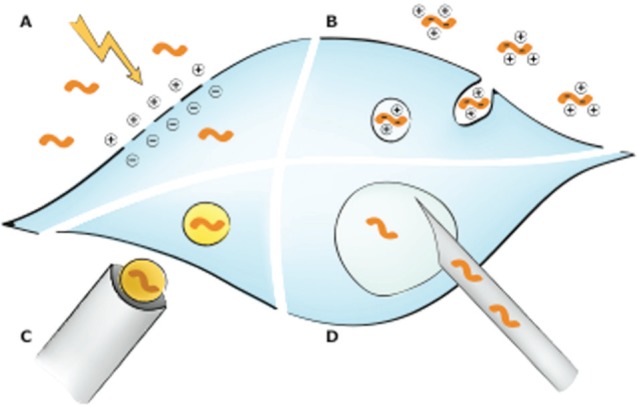
General gene delivery mechanisms. (**A**) Electroporation. During electroporation, cell membranes are destabilized allowing nucleic acid entry into the cell. (B) Reagent-based techniques. The reagents used form complexes with the negatively charged nucleic acids, which are then taken up by the cell via endocytosis. Reagents include cationic lipids, cationic polymers, and calcium phosphate. Cationic lipids form liposomes, which will fuse with the cell membrane and endosomes causing the release of the nucleic acids into the cytoplasm. Cationic polymers such as polyethylenimine condense nucleic acids. They act as a proton sponge, thus buffering acidic endolysosomes and possibly causing their rupture. How calcium phosphate/DNA precipitates are taken up and released into the cytoplasm is not well understood so far. (**C**) Biolistic particle delivery. Nucleic acid–coated gold particles are shot at target cells. (**D**) Microinjection. Via an injection needle, nucleic acids can be directly delivered into the nucleus or cytoplasm. Less frequently used methods to deliver genetic material into cells like magnetofection or laserfection as well as viral transduction methods are not displayed.

The cell membrane acts as a primary barrier to the entry of macromolecules into cells. From a biotechnological perspective, the uptake of large macromolecules, in particular of plasmid DNA and RNA oligonucleotides, is highly desirable for gene manipulation purposes. Accordingly, various methods have been developed that allow the introduction of exogenous material to the cell. This process is generally referred to as *transfection*. There are two main categories for transfection. The first is reagent-based methods including lipofection, calcium phosphate precipitation, cationic polymers, DEAE-dextran, activated dendrimers, and magnetic beads (**Table 1**). Reagent-based transfection methods use cellular uptake processes such as endocytosis and macropinocytosis or simply fusion of liposome particles with the plasma membrane (**Fig. 1**). The other method is instrument based including biolistic technologies, microinjection, laserfection/optoinjection, and electroporation (**Table 1**). Virus-based methods are generally termed *transduction*, which is highly efficient for most cell types, including stem cells.^1^ Transduction is mechanistically and technically very distinct from direct gene transfer by transfection as it requires the production of encapsulated DNA or RNA in virus-like particles by an intermediate step and will not be discussed here in detail. In the scope of this review, we will focus on transfection for gene manipulation and large-scale high-throughput screening (HTS) studies.

**Table 1.** Transfection Methods.

Method	Mechanism	Advantage	Disadvantage	High-Throughput Screening?
Chemical (e.g., calcium phosphate)	Endocytosis or phagocytosis	Cheap; high efficiency	Not applicable to all cell types; reagent consistency; does not work in RPMI medium	Yes
Cationic lipids (e.g., lipofectamine)	Lipids merge with membrane	Cheap; high efficiency	Not applicable to all cell types	Yes
Cationic polymers (e.g., PEI, DEAE-dextran, dendrimers)	Endocytosis or macro-pinocytosis	Cheap	Not applicable to all cell types	Yes
Magnetofection	Magnetic force	Rapid; high efficiency	Adherent cells only	No
Electroporation	Membrane pores	High efficiency	Cost; cell toxicity	Yes
Biolistic particle delivery		Targeted delivery; cell type independent	Low efficiency; cost	No
Micro-injection			Laborious; cost; technically demanding	No
Laser-fection, optofection	Laser light to permeabilize cell membrane	Works for many cell types and substances	Cost; adherent cells only; technically demanding	No
Soaking		Easy	*Drosophila* only	Yes
Feeding	Sid-1 transporter	Easy	Worms	Yes

## Gene Manipulation

The human being contains a blend of many humors. When the humors are balanced, the human being is healthy, but when they are unbalanced or improperly mixed and one is more concentrated than the other, pain and disease is the result.^2^

—Hippocrates: *On Ancient Medicine*

To identify the function of a gene in a cellular process, the most common approach is to unbalance gene expression and observe the resulting effects. This can be achieved by gain-of-function approaches such as cDNA overexpression or loss-of-function methods such as RNA interference (RNAi). The ultimate goal is to enhance or reduce, respectively, the activity of a single gene or protein in cells in order to study their contribution to a cellular process or disease. Various methods that allow gene manipulation (**Fig. 1**) are described in more detail below.

### Loss-of-Function Screening

#### RNAi

Screening for loss-of-function phenotypes typically requires the knockout or knockdown of an endogenous gene or protein. This can be achieved by transfection of short interfering (si) RNA oligonucleotides or short hairpin (sh) RNA–based plasmid vectors. RNAi has emerged as a powerful tool for loss-of-function screening, not least because siRNA libraries are commercially available in several customizable formats and sizes. Genome-wide screens using siRNA libraries have provided valuable insights into cellular factors required for virus infection,^3^ endocytosis,^4^ and regulation of mitosis,^5^ just to name a few. Both siRNA and shRNA use double-stranded RNA molecules that lead to sequence-specific degradation of mRNA in the target cells.^6^ When sequences within the genome show partial complementarity to the seed region (nucleotides 2–8), the siRNA/shRNA can exert quite significant off-target effects.^7,8^ Computational methods and chemical modifications to the siRNA oligonucleotides have made significant improvements to reduce off-target silencing.^9^ The efficiency of RNAi also depends on multiple other parameters such as the half-life of the mRNA, the amount of mRNA, and the localization in the cell.

Efficient delivery of the siRNA or shRNA to the cytoplasm is a prerequisite for efficient knockdown of the target gene. In *Caenorhabditis elegans*, dsRNA is taken up through a membrane channel transporter called sid-1.^10^ In *Drosophila*, uptake of dsRNA is mediated by endocytic or phagocytic routes.^11^ In most other cells and organisms, large charged molecules do not cross the cell membrane easily, and specialized methods for gene delivery are required. A common siRNA delivery method is electroporation, which will be discussed in detail further below.

#### Genome editing

Alternative methods for loss-of-function screening rely on genome-editing technologies such as zinc finger nucleases, transcription activator-like effector nucleases, or clustered regularly interspaced short palindromic repeats (CRISPR). Of these, CRISPR/Cas has emerged as a powerful tool for the generation of large-scale HTS libraries.^12^

CRISPR are DNA loci with repetitive sequences that act as a primary defense against invading pathogens in some bacteria and most archaea. This acquired immunity provides defense against plasmids and phages through the activation of CRISPR-associated Cas genes, resulting in endonuclease-mediated cleavage of the foreign DNA sequence.^13^ The CRISPR/Cas system has recently been adapted to become an efficient tool for genome engineering.^14–16^ This simple genome-editing system allows the generation of gene knockouts/knockins, transgenes, and modification in a much shorter time frame than alternative methods. The CRISPR/Cas system requires three components: the Cas endonuclease, a crRNA that targets the Cas nuclease to the desired genomic location, and a tracrRNA that mediates complex formation of the crRNA with the Cas protein. Similar to siRNA, a short RNA sequence of 20 nucleotide length provides sequence specificity. There are conflicting studies reporting variable degrees of off-target effects,^17–19^ with some labs claiming that off-target effects are almost absent when the guide RNA (gRNA) are properly designed. Several groups have recently reported the generation of large-scale libraries with sequence-specific gRNAs for high-throughput screening applications.^20–23^ To date, only libraries of pooled lentiviral vectors exist, which have limited use in positive or negative selection screens. Efforts are under way to generate arrayed libraries, which will broaden the range of potential applications in high-content and high-throughput phenotypic screening.

### Gain-of-Function Screening

#### cDNA overexpression

Arrayed cDNA libraries are available from various commercial vendors for human and mouse genes. Some libraries are available as expression-ready clones, often with attached tags for detection such as Flag, myc, or green fluorescent protein (GFP). Others, such as the ORFeome collection, are available as gateway clones that allow easy manipulation of the vector and introduction of modifications such as tags or other fusion proteins. To date, almost the entire human and mouse genome are available from commercial vendors (e.g., Origene, ThermoFisher), although a few clones and variants remain to be identified and added to these collections. Most libraries are partially sequence verified. The principle of cDNA expression relies on a deliberate increase in copy numbers for one gene. Historically, cDNA expression cloning has been highly successful in identifying virus and growth factor receptors.^24^ For organisms in which arrayed libraries are not commercially available, they can be generated by cDNA amplification of mRNA, as it has been historically done to create the so-called “million clone” libraries. Some of the caveats of cDNA overexpression include the difficulty of controlling the expression level of the encoded proteins, promoter interference, and mosaic expression in cells or tissues. Nonetheless, cDNA expression libraries are very powerful tools to study gene function in a large-scale screening format, as well as to complement and rescue siRNA loss-of-function experiments. Furthermore, bacterial artificial chromosome expression libraries may help overcome some of the mentioned artifacts associated with cDNA expression.^25^

#### CRISPR activation

Recently, an alternative to cDNA overexpression for gain-of-function studies that uses CRISPR-mediated genome editing has been developed. This approach relies on the activation of specific target genes through DNA-guided transcriptional enhancers,^26–31^ and it has been used with genome-wide CRISPR activation libraries. The group of Jonathan Weissmann generated a library of gRNAs both for inactivation and activation of transcription.^32^ Feng Zhang’s group synthesized 70,290 gRNAs targeting all human RefSeq coding genes.^33^ Most gRNA libraries require transfection of a plasmid DNA, and it remains to be seen whether gRNA oligonucleotide transfection can also serve to mediate CRISPR/Cas genome editing.

All of the above-described gene manipulation approaches including si/shRNA, cDNA, and CRISPR-based methods require efficient delivery of plasmid DNA or RNA oligonucleotides to cells by transfection.

## Gene Delivery Methods

The main methods for gene delivery are based on chemical or lipofection reagents (**Table 1**). Chemical methods are particularly cheap and generally achieve high transfection efficiencies in standard transformed cell lines such as HEK293 or COS cells. However, these methods work much less efficiently in primary cells and a large number of cancer cell lines. Lipofection-based methods are very efficient for introduction of foreign material into a large number of cell types. There are some limitations regarding the transfection of primary cells. Other methods such as ballistic transfection, magnetofection, and microinjection are less commonly used as they require a specialized technical setup and high levels of training. These methods are generally not amenable to HTS approaches as they cannot be standardized into robust automated assays. Electrical methods work very well for a large number of cell types. Recently, electroporation instruments have become available to enable the standardized electroporation in multiwell plates, thus making it possible to use this method in HTS applications.

In general, transfection methods impose a stressful condition to cells. Common artifacts resulting from transfection are the induction of autophagy in many cell lines,^34^ interference with translation mechanisms, and an induction of an interferon (IFN) response on shRNA transfection in mammalian cells.^35^ For instance, in bovine bone marrow–derived macrophages, most lipofection-based methods resulted in an induction of an IFN response, whereas electroporation was not only superior in terms of transfection efficiencies but also very importantly did not induce an IFN response.^36^ It remains to be shown whether electroporation in general presents fewer side effects on cellular processes, although it should be noted that electroporation can result in some degree of toxicity and associated phenomena.

### Electroporation

The use of electrical impulses on cells dates back to early studies by Neumann and Potter on mouse myeloma cells.^37–39^ More recently, electroporation has been recognized as a powerful method to deliver plasmid DNA and siRNA oligonucleotides into cells that are otherwise difficult to transfect. For instance, electroporation has been successfully used to deliver cDNA plasmids and siRNA oligonucleotides into T cells,^40^ hMSC, NHA, NDHF-neo, human umbilical vein endothelial cell (HUVEC), DI TNC1, RPTEC, PC12, and K562 cells.^41^

The three main effects of applying an electrical field to cells—depending on the strength of the electric field—are (1) the formation of pores in the membrane(s), (2) the movement of cells in dielectrophoresis, and (3) cell fusion. The formation of pores will facilitate uptake of macromolecules in cells.

The basic principle underlying all of these experiments is that brief, high-intensity electrical pulses will create transient pores in the membrane of cells. When the electrical pulses are turned off, the membranes reseal, which is essential for survival of the cells. It can be imagined that opening and resealing provide a condition wherein large macromolecules can enter and exit the leaky cell. Thus, the duration and spacing of pulses, as well as the intensity, are crucial for high levels of uptake of macromolecules in cells. In addition, the geometry of the electrodes will determine the electrical field applied to a cell monolayer or three-dimensional structure and is therefore critical for successful gene delivery. The main parameters that determine electropermeability are the following:

- cellular factors such as cell density, cell architecture, and cell biochemistry;
- physicochemical factors such as temperature, pH, osmolarity, and ionic concentration of the buffer used; and
- electrical parameters such as electrical field strength, voltage, pulse length, pulse number, and electrode geometry.

The selection of appropriate buffers used for electroporation will also affect the efficiency of gene delivery. Main parameters for selecting the right buffer are osmolarity and ionic strength, while maintaining the highest possible survival rate. Buffers that mimic intracellular ionic strength have been generally recommended,^42^ but in general, most standard media work reasonably well. Improvements to media compositions aim to enhance cell survival postelectroporation so that harsher electroporation parameters can be used. Electroporation conditions for each cell type will therefore be slightly different, but there are some general guidelines and principles that we will outline below.

### Principle

**Figure 2.**
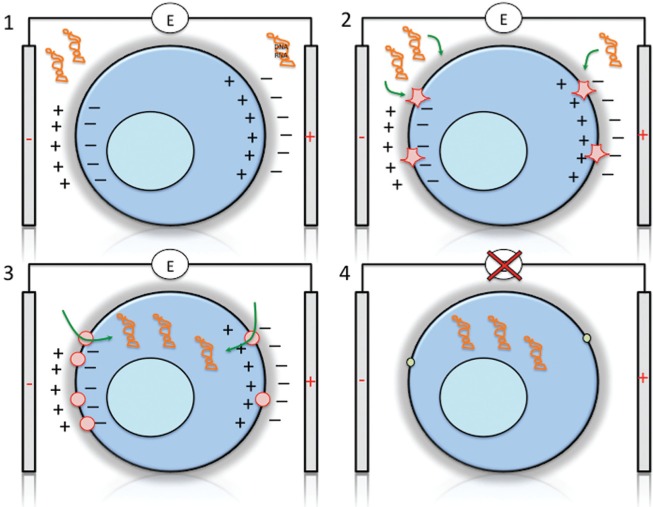
Electroporation of cells. Electroporation occurs through four main steps: (1) polarization of the cell, (2) rapture of the membrane creating nanopores, (3) entry of the macromolecules, and (4) resealing of the membrane. (**1**) Application of short electrical pulses will result in membrane charging, creating an electrical field and resulting in polarization of the cell. The strong electrical field will result in structural rearrangements of the membrane, creation of water-filled membrane structures (“aqueous pores”) and “nanopores” with a size of more than 1 nm that allow ionic transport. (**2**) Larger pores are formed in the membrane that allows influx of macromolecules such as DNA or RNA. Generally, more pores are formed at the site facing the negative electrode. (**3**) Large macromolecules can enter the cell. The negative charge of DNA/RNA can act as a drag to enhance uptake, although, on the other hand, positive ions such as calcium can enhance proximity to the negatively charged membrane prior to uptake. (**4**) Electroporation is reversible, and once the electric field is switched off, the membrane has the capacity to reseal and keep the macromolecules inside the cell. Resealing occurs on a much longer time frame (minutes to hours), whereas pore formation can occur within milliseconds. Low temperature can enhance resealing, although this may not be practical for eukaryotic cells in some applications.

The basis for all electrochemical manipulations of cells is the formation of a polarized cell in an electric field (**Fig. 2**). This occurs as a consequence of the interaction of an electrical field with charges on the cell surface and interior of the cell. Accordingly, the cellular membrane will impose a restriction on the movement of charges in an electrical field and thus lead to polarization of the cell when placed between two electrodes. This polarization of the cell can result in motions in the cytoplasm leading to structural rearrangements or mechanical fracture. Cell membranes have low polarizability with a dielectric constant of about 2 and low conductivity (~1 nS/cm), whereas the surrounding media have a high dielectric constant (around 80) and high conductivity (about 0.1 S/cm).^43^ The application of an electrical field will result in high movement of ions in the surrounding media and accumulation at the membrane surface, thus leading to polarization. This in turn will create an electrical field inside the cell that can be of much higher strength than the field in the surrounding media and thus lead to the formation of pores resulting in discharge of the charges at the membrane. The basic relationship to calculate the voltage across a membrane in a spherical object is estimated as

(1) $$V=V_{m}[1-\exp(-\tau /t_{p})]$$

where *V*_m_ is the maximum transmembrane voltage, τ is the duration of the pulse, and *t*_p_ is the polarization time.^44^ For spherical membranes of radius *R, V*_m_ and the time constant *t*_p_ are

(2) $$V_{m}=1.5\;ER\;\cos\delta$$

(3) $$t_{p}=RC_{m}\left(r_{i}+\;0.5r_{o}\right)$$

where δ is the angle between the electric field, *E*; the radius vector, *C*_m_, is the membrane capacitance; *r*_i_ and *r*_o_ are resistivities inside and outside the cell; and the membrane conductance is neglected. In this equation, the cell radius directly correlates with the strength of the electrical field that needs to be applied. Larger cells can be electroporated with smaller strength *E* than smaller cells. For instance, at the poles (δ = 0), the induced transmembrane voltage is maximal. Another important parameter is the charging time *t_p_*, which should always be smaller than the pulse duration τ, as nanosecond pulses with high electric fields typically result in cell killing.^45^ A detailed strength-duration map has been formulated that predicts the outcome of varying these parameters.^46^ For instance, cells with higher resistance and larger cell radius require longer pulse duration times.

The above equations consider cells as spherical objects for simplicity. The transmembrane voltage for a nonspherical, ellipsoid, or cylindrical cell in an arbitrarily oriented position can be described by

(4) $$V_{a}=1/\left(1-n_{a}\right)aE$$

where *a* stands for the semiaxis oriented in field direction and *n*_a_ for the depolarizing factor along semiaxis *a*.^47^ More complex cell shapes are described elsewhere.^48^

Membranes are electroporated when the transmembrane voltage *V* exceeds a critical threshold, which is typically in the range of several hundred mV to 1 to 2 V. Generally, an increase in the pulse duration equates to a decrease in the voltage required. It should be noted that the application of electrical fields results in heating of the sample, and cooling has been recommended to improve cell survival. Therefore, increases in temperature (e.g., due to increased field strength) will require shorter pulse duration.^46^

Another important consideration is the kinetics of pore opening and resealing. Typically, pore formation occurs within microseconds and initial recovery of the cell membranes within milliseconds. Complete recovery may take much longer (i.e., seconds, minutes, or even hours). Longer pulses produce larger pores, and very long pulses and/or high voltages will lead to irreversible membrane damage. It has been noted that pore formation and electroporation of large molecules into cells can be asymmetric with higher permeation on the one electrode side, an effect that is dependent on the salt concentration of the buffer used.^49^

For negatively charged molecules, such as siRNA oligonucleotides, it was noted that the electric field acts both to permeabilize the cell membrane as well as to provide an electrophoretic “drag” of the negatively charged siRNA molecule from the bulk phase into the cytoplasm.^50^ Therefore, there may be an advantage for delivery of charged molecules by electroporation. Similarly, it was noted that electroporation of crude DNA preparations were more efficiently transferred to cells than purified CsCl_2_ preparations because of a carrier effect of the contaminating bacterial RNA.^51^

The first demonstration that electroporation could be used for transfection and expression of a foreign gene was made in 1982, and the term *electroporation* was coined.^38^ Since then, electroporation has been used for multiple cell types, including cell lines, primary cells, plant cells, and single-cell organisms. Electroporation can also be used to introduce genes in whole tissues or organisms.

The application of electrical pulses to cells can also lead to another phenomenon called *electrofusion*, whereby two adjacent cell membranes fuse with each other, thereby generating multinucleated cells. For electrofusion to occur, the membranes must be in close proximity. The most efficient way to ensure whether either fusion or permeabilization occurs is by reducing the cell density in a sample preparation. As low cell density is often associated with a higher vulnerability of the cells to stress, this poses a challenge for efficient electroporation protocols.

## In Vivo Electroporation

An exciting and growing area is the use of electroporation in vivo for delivery into tissues and whole animals. Electroporation has been achieved in complex models. Examples include the electroporation of DNA in mouse testis,^52^ the subretinal space,^53^ and zebrafish forebrain.^54^ Other applications include the delivery of shRNA plasmids and siRNA oligonucleotides in skeletal muscle,^55^ kidney glomeruli,^56^ the developing cerebella,^5^, and in vivo solid tumors.^58^

When using electroporation in tissues such as solid tumors, the electrical field and transmembrane voltage will be dependent on the tissue architecture and microenvironment of the cells.^59,60^ For instance, it was observed that the electric field in cells surrounded by other cells can be reduced by as much as one-third compared with single cells in suspension.^61^ In general, efficiency of electrotransfer in tissues is relatively low with only a few percentage of transfected cells^62^ that are mostly found in the periphery of a tumor.^63^ To gain insight into these phenomena, electroporation in simpler three-dimensional (3D) model systems such as spheroids has been studied.^64^ Interestingly, although interior cells in such spheroid models can be efficiently permeabilized, the uptake of large macromolecules such as DNA is generally limited to the outer layers, probably because of inefficient transport across longer distances.^61,64^ It was proposed that low voltage but long pulses may be able to electrophoretically push the DNA toward the center of a spheroid or tissue.

Typically, needle electrodes are used for in vivo electroporation, sometimes in combination with ring-shaped or plate electrodes. The overall arrangement of the electrodes will greatly affect the electroporation efficiency. Recently, a three-electrode system was designed for efficient electroporation in utero of neural cells.^65^ The triple-electrode arrangement allowed testing changes in electrical field distribution, which overall enhanced the transfection efficiency in rat Purkinje cells.

Electroporation in tissues and 3D model systems is an intense area of research and will find applications in clinical and preclinical use.

## Applications of Electroporation in HTS

The instruments for electroporation require only two components: a high-voltage pulse generator and two electrodes embedded in the cell suspension. **Table 2** summarizes electroporators for multiwell plate format from various sources.

**Table 2.** Selected Instruments for High-Throughput Electroporation in Multiwell Formats.

Instrument	Model	No. of Parallel Processing	Plate Type	Comments/Web Site
Cellectricon	Cellaxess Elektra	96	384 well	www.cellectricon.com
Lonza/Amaxa	Nucleofector	1	96 or 384 well	www.lonza.com
Harvard Apparatus	BTX830	8	96 well	www.btxonline.com
BIORAD	GenePulser MXcell	Flexible	96 well	www.bio-rad.com
Primax	iPorator-96	96	96 well	www.primaxbio.com
Ambion	siPORTer96	1	96 well	

There are several requirements for electroporation in a high-throughput format: first and foremost, the instrument has to enable an efficient processing of multiple samples in a short time frame, requires minimal manual steps involved in electroporation, and has to be cost-effective and minimize volumes of the transfected material. Typically, relatively large volumes are required for cuvette-type transfections, and therefore, multiple vendors have designed microplate electroporation instruments. Most of these use sequential processing of samples (siPorter96, Nucleofector) and thus require a relatively long time for completion from the first to the last well. The BTX allows processing of eight samples in parallel, and the Cellaxess and Primax iPorator96 are instruments that allow processing of 96 samples in parallel. Only two instruments allow transfection in 384-well plate format (Cellaxess and Nucleofector 4D). Another factor is that because of the arrangement of the electrodes, most instruments enable transfection of cells in suspension (with the exception of the Cellaxess), making this impractical for applications in which adherent cell differentiation is required (e.g., induced pluripotent stem cell [iPS]–derived neurons).

To date, very few applications of electroporation in high-content and phenotypic screening have been published. Although a number of electroporation devices are available for the use in 96-well and 384-well plate format, work has been focused on establishing and optimizing protocols for the electroporation of otherwise difficult-to-transfect cells. In particular, it has been proposed that electroporation can facilitate phenotypic screening using siRNA and cDNA libraries in difficult-to-transfect cell lines such as neurons, stem cells, and macrophages.

Primary neuronal cultures as well as neurons derived from iPS cells are an essential model to study molecular and biochemical processes involved in neurogenesis, neuronal function, and plasticity as well as neurodegenerative diseases. Postmitotic and differentiated neurons, however, are challenging to transfect. Their elongated neurites and their sensitivity to physical stress, alterations in temperature, pH shifts, and changes in osmolarity contribute to low transfection efficiencies (reviewed in refs. 66–68). Thus, protocols are needed that boost efficiency while keeping toxicity to a minimum. Optimization protocols for potential RNAi and cDNA library screening were performed using a variety of instruments, which are based on two main approaches: the electroporation of cells in suspension directly on the isolation of neurons or neuronal progenitor cells and the electroporation of adherent neurons in culture. Examples for transfection of neurons in suspension include nucleofection with the Lonza Nucleofector and transfection with the system provided by BTX Havard Apparatus. Optimization protocols for both instruments are available. For example, successful transfection of rat neurons has been done using the BTX system.^69^ Hutson et al.,^70^ who isolated cerebellar granule neurons from rat pups (P7-9), transfected the obtained neuronal suspension with cDNA using a 96-well electroporation plate, the HT-200 plate handler, and the ECM 830 square-wave pulse generator by BTX Harvard Apparatus and plated the transfected neurons thereafter. The pulse parameters of one pulse at 300 V and 1 ms length resulted in 28% transfection efficiency and 51% viability. A similar study was performed by Buchser et al.^69^ using the same setup and comparable high pulse voltage (340 V) and short pulse length (900 µs). Another protocol, describing the transfection of freshly isolated embryonic rat and mouse hippocampal neurons with the Lonza Nucleofection 96-well shuttle system was published by Zeitelhofer et al.^71^ The Nucleofection system also uses short high-voltage pulses to ensure high transfection efficiency.

Electroporation of adherent neurons provides several advantages such as enabling the use of in-plate differentiation protocols, reduced toxicity, and the ability to perform high-content imaging without subjecting the cells to stressful detachment and reseeding. The Cellaxess Elektra system enables transfection of adherent neuronal cultures in a 384-well format. It was shown that the capillary electroporation concept of the Cellaxess Elektra allows the successful transfection of hippocampal and cortical neurons, which were isolated from rat brains (E18) and subsequently cultured for 5 d to allow axon and dendrite development.^72^ In this study, two short 2 ms pulses at 350 V resulted in 30% of GFP-positive neurons when a GFP plasmid was used for transfection and a significant reduction of GFP when siGFP was co-transfected. Cell viability was not impaired compared with nonelectroporated samples. Higher voltage pulses yielded higher transfection efficiencies of up to 50% with viabilities still greater than 85%.

Besides neurons, human embryonic stem cells (hESCs) have been proven difficult to transfect. hESCs are derived from blastocyst-stage embryos, are able to proliferate continuously. and can differentiate into any cell type.^73,74^ Other studies have shown that the transfection efficiency with chemical reagents such as Lipofectamine, Fugene, or Exgen 500 is very low.^75,76^ Electroporation protocols result in a slightly more efficient transfection, but parameters need to be optimized to avoid substantial cell death. Nucleofection seems to deliver good transfection and viability results and was optimized for the use in 96-well plate format, as shown by Moore et al.^77^ They transfected various undifferentiated hESC lines with only a loss of about 25% due to cell death. The variability of the transfection efficiency between experiments was high, ranging from just greater than 20% to three-quarters of cells being transfected. The nucleofection shuttle system was also used in a microRNA (miRNA) screen to identify miRNAs involved in the regulation of hematopoiesis.^78^ For this screen, H9 hESCs were differentiated into common myeloid progenitor cells. Cells were then transfected with the pre-miR miRNA precursor library-human V3 and cultured for 4 to 5 d in 96-well plates before flow cytometry analysis revealed the impact of various miRNAs on hematopoiesis.

Differentiated macrophages have also been shown to be possible target cells of siRNA screening with the 96-well shuttle system.^79^ A pilot screen was described in which a set of siRNAs was investigated regarding their ability to regulate lipopolysaccharide-induced cytokine production. In this preliminary study, fluorescein isothiocyanate–labeled siRNA was used to show the high transfection efficiency.

Another study used nucleofection in otherwise hard-to-transfect HUVECs to identify genes that regulate cell viability.^80^ The viability assays were performed 72 h after nucleofection with the Dharmacon Human siGenome SMART pool siRNA libraries for protein kinases and cell cycle regulation.

Overall, electroporation in high-throughput format is feasible but has not been taken up by the scientific community. The main reason for this is the associated cost. The business strategy for all commercially available instruments relies on the purchase of proprietary buffers and plates at a very high cost. This makes the application of all commercial electroporation instruments impractical for large-scale screening. This is highly surprising as it defeats the purpose of an instrument that was built for large-scale applications.

For this reason, several academic laboratories have explored the design and engineering of electroporation systems that circumvent these problems. Design of a customized electroporator is relatively simple, as it only requires two main components: a high-voltage pulse generator and two electrodes embedded in a cell suspension. The two main types of customized systems are (1) flow electroporation chips and (2) suspended drop-based electroporation systems in a 96-well microplate format.

Flow-based systems enable a rapid transfection of a large number of cells in a continuous or semi-continuous flow.^81^ Generally, these systems are used when a large number of cells are needed, for instance, in clinical research. On the contrary, when a large number of samples are interrogated, microplate-based electroporation systems are required.

A 12-well electroporation prototype plate was developed that uses interdigital electrode arrays for efficient electroporation of siRNA and plasmid DNA in cell lines and primary cells, and a 96-well design that is compatible with standard microplate formats was proposed.^82^ Another system that allows high-throughput electroporation enabling parallel electroporation of spotted siRNAs in microwell arrays has been described recently.^83^ The surface of the arrays is functionalized with amine groups so that coupling of siRNA molecules and parallel electroporation in one microwell chamber is possible. siRNAs were spotted in a 9 × 9 array using automated noncontact inkjet printing, and cells were electroporated quickly after seeding into the chamber. In principle, this system allows a high-throughput electroporation of thousands of siRNAs in a miniaturized array format.

A suspended drop-electroporation system has been presented that allowed high-efficiency transfection of cell lines and primary cells in a microplate format.^84^ This system uses suspended electrode pairs in a 96-well microplate format for top loading of samples, thus allowing the use of robotic liquid handling and parallel processing of 96 samples. Buffers that can be used are very cheap standard media. This system enables electroporation of DNA, siRNA, and small molecules at a low cost in a very short time frame.

A similar device for top loading of cells into through-holes of a pair of gold-coated electrodes was described recently.^85^ In this system, the 96-well electrode array is placed on top of standard microplates, allowing the ejection of cells into the bottom plate by straightforward addition of cell media. All 96 samples are electroporated in parallel, and each well can be addressed separately, representing a very flexible and modular system. Such systems will enable the development of simple 96-well electroporation plates as cheap consumables that can be placed on top of any receiver plate. This will be a step forward toward realizing a modular approach for high-throughput electroporation. The user could then select a receiver plate of choice suitable for biochemical assays or with optically clear bottom for high-content screening. It can be expected that these systems will replace the traditional plate-based inflexible formats of current commercial vendors.

Electroporation has historically been considered as the last straw when other transfection methods have failed. Although electroporation instrumentation for HTS is now readily available, access to microplate-based electroporators and the high consumable cost are still major hurdles to implement this in the wider community. Given that this is such a powerful method, maybe it is time to rethink the business strategy in this area.

We have observed that most major cell types including primary cells can be easily transfected by electroporation. Given the higher physiological relevance of these models, it can be expected that the implementation of large-scale electroporation will increase in the coming years.

Interesting areas to watch are the further development of high-throughput electroporation devices that are either based on flow chips or suspended drop systems. Also, the use of flexible material for supporting the electrodes such as parylene allowing embedding of target cells/tissues within the electrodes will become extremely valuable for physiologically relevant systems and in vivo electroporation.^86^

It can be expected that these and other developments will facilitate large-scale screening in the future. Especially for the emerging CRISPR technology, this will be important as most CRISPR-based methods to date require plasmid-based transfection or transduction.
